# Propiverinium picrate

**DOI:** 10.1107/S1600536809022995

**Published:** 2009-07-01

**Authors:** Jerry P. Jasinski, Ray J. Butcher, Q. N. M. Hakim Al-Arique, H. S. Yathirajan, B. Narayana

**Affiliations:** a Department of Chemistry, Keene State College, 229 Main Street, Keene, NH 03435-2001, USA; b Department of Chemistry, Howard University, 525 College Street NW, Washington, DC 20059, USA; c Department of Studies in Chemistry, University of Mysore, Manasagangotri, Mysore 570 006, India; d Department of Studies in Chemistry, Mangalore University, Mangalagangotri 574 199, India

## Abstract

The title compound [systematic name: 4-(2,2-diphenyl-2-prop­oxyacet­oxy)-1-methyl­piperidin-1-ium picrate], C_23_H_30_NO_3_^+^·C_6_H_2_N_3_O_7_^−^, crystallizes as a salt with one cation–anion (propiverinium picrate) pair in the asymmetric unit. A significant number of conformational changes are observed between the crystalline environment of this cation–anion salt and that of a density functional theory (DFT) calculation of the geometry-optimized structure. The angle between the dihedral planes of the two benzyl rings in the propiverinium cation increases by 14.4 (0)° from that of the crystalline environment. The dihedral angles between the mean planes of each of the benzyl rings and the mean plane of the piperidine increase by 2.0 (8) and 12.3 (5)°. The angles between the mean plane of the acetate group and the mean planes of the inter­connected piperidine group and the two benzyl rings decrease by 0.2 (1), 7.4 (6) and 3.2 (2)°, respectively. The mean plane of the phenolate group in the anion changes by +22.6 (9), +22.1 (1) and −2.8 (6)° from the mean planes of the piperidine and benzyl rings in the cation, respectively. In the crystal, a bifurcated N—H⋯(O,O) hydrogen bond and a weak C—H⋯π ring inter­action help to establish the packing. The two O atoms of the *p*-NO_2_ group are disordered with occupancies 0.825 (10):0.175 (10).

## Related literature

For related structures, see: Bindya *et al.* (2007 ▶); Harrison, Bindya *et al.* (2007 ▶); Harrison, Sreevidya *et al.* (2007 ▶); Swamy *et al.* (2007 ▶) Yathirajan *et al.* (2007 ▶). For background, see: Chapple *et al.* (2008 ▶); Jünemann *et al.* (2006 ▶); Madersbacher & Gramatté, (2006 ▶); Matsushima *et al.* (1997 ▶); Noguchi & Masuda, (1998 ▶); Okada & Sengodu, (1998 ▶); Rong *et al.* (1999 ▶). For density functional theory (DFT), see: Becke (1988 ▶, 1993 ▶); Frisch *et al.* (2004 ▶); Hehre *et al.* (1986 ▶); Lee *et al.* (1988 ▶); Schmidt & Polik (2007 ▶); Szumma *et al.* (2000 ▶). For puckering parameters, *see*: Cremer & Pople (1975 ▶).
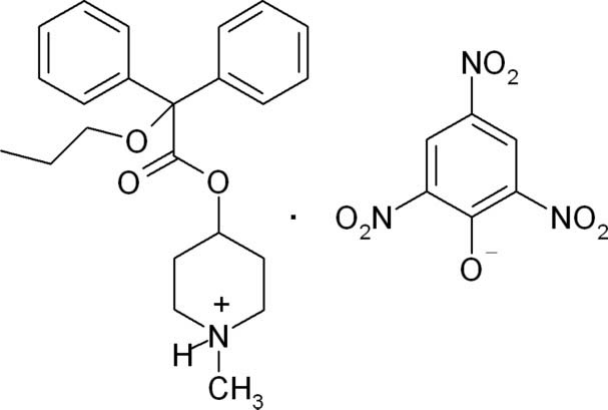


## Experimental

### 

#### Crystal data

C_23_H_30_NO_3_^+^·C_6_H_2_N_3_O_7_^−^
*M* *_r_* = 596.59
Triclinic, 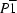

*a* = 8.9379 (4) Å
*b* = 9.2885 (4) Å
*c* = 18.0750 (7) Å
α = 97.652 (3)°
β = 97.630 (3)°
γ = 104.301 (4)°
*V* = 1419.54 (10) Å^3^
*Z* = 2
Mo *K*α radiation
μ = 0.11 mm^−1^
*T* = 110 K
0.55 × 0.35 × 0.27 mm

#### Data collection

Oxford Diffraction Gemini R CCD diffractometer
Absorption correction: multi-scan (*CrysAlis RED*; Oxford Diffraction, 2007 ▶) *T*_min_ = 0.910, *T*_max_ = 0.972
18429 measured reflections
9328 independent reflections
6353 reflections with *I* > 2σ(*I*)
*R*_int_ = 0.022

#### Refinement

*R*[*F* ^2^ > 2σ(*F* ^2^)] = 0.046
*wR*(*F* ^2^) = 0.126
*S* = 1.03
9328 reflections
397 parameters
24 restraints
H-atom parameters constrained
Δρ_max_ = 0.39 e Å^−3^
Δρ_min_ = −0.31 e Å^−3^

### 

Data collection: *CrysAlisPro* (Oxford Diffraction, 2007 ▶); cell refinement: *CrysAlisPro*; data reduction: *CrysAlisPro* (Oxford Diffraction, 2007 ▶); program(s) used to solve structure: *SHELXS97* (Sheldrick, 2008 ▶); program(s) used to refine structure: *SHELXL97* (Sheldrick, 2008 ▶); molecular graphics: *SHELXTL* (Sheldrick, 2008 ▶); software used to prepare material for publication: *SHELXTL*.

## Supplementary Material

Crystal structure: contains datablocks global, I. DOI: 10.1107/S1600536809022995/at2814sup1.cif

Structure factors: contains datablocks I. DOI: 10.1107/S1600536809022995/at2814Isup2.hkl

Additional supplementary materials:  crystallographic information; 3D view; checkCIF report

## Figures and Tables

**Table 1:** Hydrogen-bond geometry (Å, °)

*D*—H⋯*A*	*D*—H	H⋯*A*	*D*⋯*A*	*D*—H⋯*A*
N1*B*—H1*BD*⋯O1*A*	0.93	1.81	2.6276 (12)	145
N1*B*—H1*BD*⋯O2*A*	0.93	2.33	3.0537 (15)	135
C20*B*—H20*B*⋯*Cg*2	0.99	2.77	3.7553 (13)	173

*Cg*2 is the centroid of the C5*B*–C10*B* ring.

**Fig. 1.**
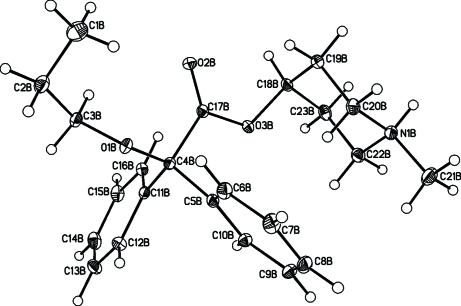
Molecular structure of the C23H30O3N+ cation showing atom labeling scheme and 50% probability displacement ellipsoids.

**Fig. 2.**
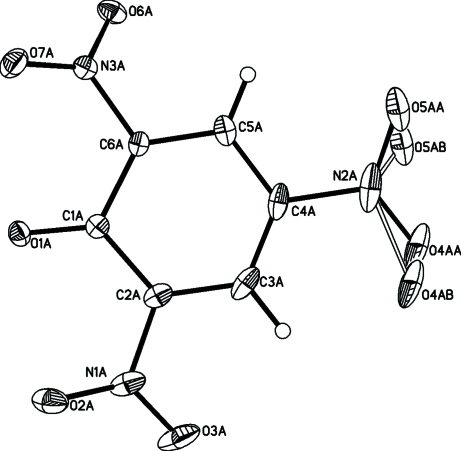
Molecular structure of the C6H2N3O7- anion showing atom labeling scheme and 50% probability displacement ellipsoids. Both components of the disordered nitro group are displayed [O4AA(0.825 (10))-N2A—O5AA(0.825 (10)) & O4AB(0.175 (10))-N2A—O5AB (0.175 (10))].

**Fig. 3.**
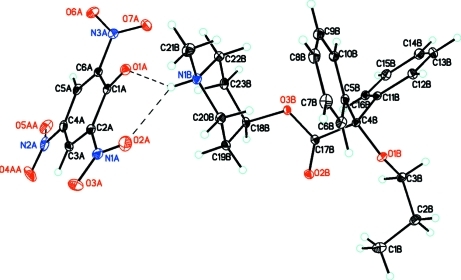
Molecular structure of the C23H30O3N+, C6H2N3O7- cation-anion pair showing the atom labeling scheme and 50% probability displacement ellipsoids. Dashed lines indicate N1B—H1BD···O1A and N1B—H1BD···O1B hydrogen bond interactions. Only the predominate nitro oxygen atoms are displayed for the disordered nitro group [O4AA(0.825 (10))-N2A—O5AA(0.825 (10))].

**Fig. 4.**
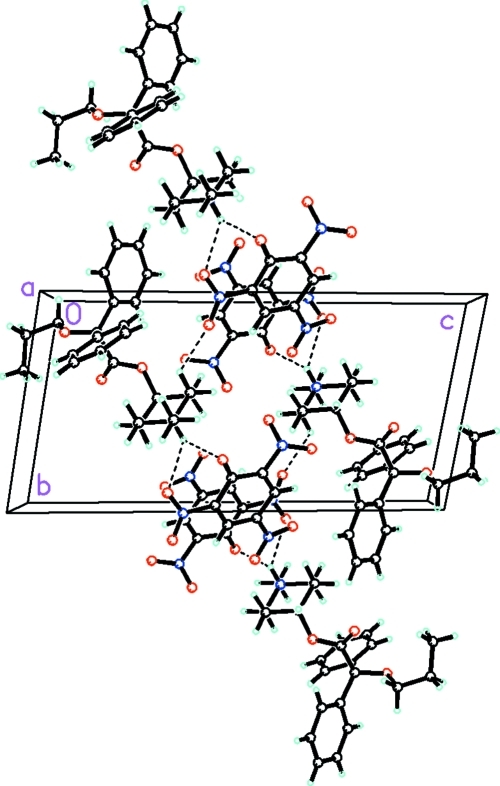
Packing diagram of the title compound, (I), viewed down the a axis. Dashed lines indicate intermolecular N1B—H1BD···O1A & N1B—H1BD···O2A hydrogen bond interactions which produces a network of infinite O—H···O—H···O—H chains arranged along the (011) plane of the unit cell.

## supplementary crystallographic information

### Comment

The title compound,C_29_H_32_N_4_O_10_, crystallizes as a salt with one cation–anion (propiverinium picrate) pair [C_23_H_30_O_3_N^+^.C_6_H_2_N_3_O_7_^-^] in the asymmetric unit. Propiverine hydrochloride, [chemical name: (1-methylpiperidin-4-yl) 2,2-diphenyl-2-propoxyacetate hydrochloride], originally developed by Schering-Plough (Okada & Sengodu, 1998; Noguchi & Masuda, 1998), is widely used in the treatment of urinary incontinence (Matsushima *et al.* 1997; Rong *et al.* 1999). Propiverine is an anticholinergic drug used for the treatment of urinary urgency, frequency and urge incontinence, all symptoms of overactive bladder syndrome. A modified release preparation is also available, taken once daily. Propiverine, a benzylic acid derivative, has been used as a urospasmolytic since 1981. It is unique in having both anticholinergic and calcium channel blocking effects. The former effects are known to suppress neurogenic detrusor contraction while the latter have a direct spasmolytic effect on the bladder. Experiments on isolated human urinary bladder strips using acetylcholine, calcium and potassium chloride and electrical fields as stimuli for contraction (Jünemann *et al.* 2006), have shown that both propiverine and tolterodine have a greater maximum inhibitory effect on bladder contraction than either atropine or oxybutynin. In the case of propiverine, calcium channel blocking effects are believed to contribute to its enhanced spasmolytic action on bladder smooth muscle (Chapple *et al.* 2008; Madersbacher & Gramatté, 2006). The crystal structures of amitriptylinium picrate (Bindya *et al.* 2007), mepazinium picrate (Yathirajan *et al.* 2007), imipraminium picrate (Harrison, Bindya *et al.* 2007), nevirapinium picrate (Harrison, Sreevidya *et al.* 2007) and desipraminium picrate (Swamy *et al.* 2007) have been reported. In continuation of our work on the picrate salts of compounds of pharmaceutical importance, this paper reports a crystal structure of the title compound, (I), C_23_H_30_O_3_N^+^.C_6_H_2_N_3_O_7_^-^, a molecular salt arising from the reaction of propiverine and picric acid.

The title compound,*C*_29_H_32_N_4_O_10_, crystallizes as a salt with two cation (propiverinium)-anion (picrate) pairs [C_23_H_30_O_3_N^+^.C_6_H_2_N_3_O_7_^-^] in the asymmetric unit cell. The propiverinium cation contains two benzyl rings whose dihedral planes are separated by 72.5 (8)° and a 6-membered piperidine group which adopts a slightly distorted chair conformation (Cremer & Pople, 1975) with puckering parameters Q, θ and φ of 0.564 (4) Å, 177.0 (6)° and 177.084 (5)°, respectively (Fig. 1). For an ideal chair θ has a value of 0 or 180°. The dihedral angles between the mean planes of each of these benzyl rings and the mean plane of the piperidine group are 0.5 (6)° and 72.8 (8)°, respectively. The piperidine group and two benzyl rings are connected by an acetate group whose mean plane makes an angle of 83.8 (8)°, 78.5 (5)° and 84.3 (1)°, with the mean planes of the piperidine group and two benzyl rings, respectively. In the picrate anion, the mean plane of two *o*-NO_2_ groups are twisted by 15.6 (6)° and 38.5 (1)° with respect to the mean plane of the 6-membered benzyl ring (Fig. 2). The two oxygen atoms in the *p*-NO_2_ group are disordered with the major components [O(4 A A) (0.825 (10)) and O5AA (0.825 (10))] making a dihedral angle of 8.9 (7)° with the mean plane of the benzyl ring. The difference in the twist angles of the mean planes of the two *o*-NO_2_ groups can be attributed to an intermolecular hydrogen bonded interaction between the piperidine group of the propiverinium cation with one of these groups, O2A—N1A—O3A, on the picrate anion, in which the O2A atom forms an intermolecular "side" hydrogen bond [N1B—H1BD···O2A] with N1B from the piperidine group (Fig. 3, Table 1). N1B also forms an intermolecular hydrogen bond with the phenolate oxygen anion, O1A, making it a two-centered hydrogen bond. This observation, when NO_2_ groups in picrate related salts form "side" hydrogen bonds resulting in a torsion angle increase of several degrees, is also seen in other similar picrate-related salts (Szumma *et al.* 2000). The difference in angles between the mean planes of the *o*-O2A—N2A—O3A and *o*-O6A—N6—O7A groups in (I) with that of the phenolate group of the picrate anion is 22.8 (5)°, a direct result of the observed N1B—H1B···O2A hydrogen bond. Crystal packing is also influenced by π-ring C—H···*Cg* intermolecular interactions with the piperidine group [C20B—H6A···*Cg*2: H···*Cg* = 2.89 Å; *X*—H···*Cg* = 173°; *X*···*Cg* = 3.7553 Å; *x*, *y*, *z*, where *Cg*2 = C5B/C6B/C7B/C8B/C9B/C10B] in the unit cell (Fig. 4).

A density functional theory (DFT) geometry optimization molecular orbital calculation (Schmidt & Polik, 2007) was performed on the C_23_H_30_O_3_N^+^, C_6_H_2_N_3_O_7_^-^ cation-anion pair of the title molecule, (I), with the *GAUSSIAN03* program package (Frisch *et al.* 2004) employing the B3-LYP (Becke three parameter Lee-Yang-Parr) exchange correlation functional, which combines the hybrid exchange functional of Becke (Becke, 1988, 1993) with the gradient-correlation functional of Lee, Yang and Parr (Lee *et al.* 1988) and the 3–21 G basis set (Hehre *et al.*, 1986). Starting geometries were taken from X-ray refinement data. The angle between the dihedral planes of the two benzyl rings in the propiverinium cation becomes 86.9 (8)°, an increase of 14.4 (0)° from that of the crystalline environment. The dihedral angles between the mean planes of each of the benzyl rings and the mean plane of the piperidine group become 2.6 (4)° and 85.2 (3)°, an increase of 2.0 (8)° and 12.3 (5)°, respectively. The angles between the mean plane of the acetate group and the mean planes of the piperidine group and two benzyl rings become 83.6 (7)° and 61.0 (9)°, 81.09°, respectively, only slightly changed from that in the crystal. A comparison of the mean planes of the phenolate group in the anion to the mean planes of the piperidine and benzyl rings in the propiverinium cation also show similar changes between the crystal and the DFT theoretical calculation [*i.e.* Phenolate-piperidine = 61.2 (1)°, crystal, *versus* 83.9 (0)° DFT; Phenolate-Benzyl = 61.2 (3)°, 33.9 (7)°, crystal *versus* 83.3 (4)°, 31.1 (1)°, DFT].

In conclusion, the significant number of conformational changes that are observed between the crystalline environment of this cation (propiverinium)-anion (picrate) salt and that of a density functional theory calculation of the geometry optimized structure support the effects of intermolecular hydrogen bonding interactions and π-ring C—H···*Cg* intermolecular interactions with the piperidine group as providing the major influence on packing effects in the crystalline environment of the title compound, propiverinium picrate,*C*_23_H_30_O_3_N^+^.C_6_H_2_N_3_O_7_^-^.

### Experimental

Propiverine hydrochloride (4.1 g, 0.01 mol) in 25 ml of methanol and picric acid (4.8 g, 0.01 mol) in 25 ml of methanol were mixed and stirred in a beaker at 318 K for two hours. The mixture was kept aside for 3 days at room temperature. The separated bright yellow salt was filtered, washed thoroughly with chloroform and dried in vacuum desiccator over phosphorous pentoxide. The salt was recrystallized from acetonitrile [m.p: 403–406 K]) by slow evaporation.

### Refinement

All of the H atoms were placed in their calculated positions and then refined using the riding model with N—H = 0.93, C—H = 0.95–0.99 Å, and with *U*_iso_(H) = 1.172–1.49*U*_eq_(C,*N*).

### Crystal data

**Table:**

C_23_H_30_NO_3_^+^·C_6_H_2_N_3_O_7_^−^	*Z* = 2
*M**_r_* = 596.59	*F*(000) = 628
Triclinic, *P*1	*D*_x_ = 1.396 Mg m^−^^3^
Hall symbol: -P 1	Mo *K*α radiation, λ = 0.71073 Å
*a* = 8.9379 (4) Å	Cell parameters from 8020 reflections
*b* = 9.2885 (4) Å	θ = 4.7–32.6°
*c* = 18.0750 (7) Å	µ = 0.11 mm^−^^1^
α = 97.652 (3)°	*T* = 110 K
β = 97.630 (3)°	Prism, pale yellow
γ = 104.301 (4)°	0.55 × 0.35 × 0.27 mm
*V* = 1419.54 (10) Å^3^	

### Data collection

**Table:**

Oxford Diffraction Gemini R CCD diffractometer	9328 independent reflections
Radiation source: fine-focus sealed tube	6353 reflections with *I* > 2σ(*I*)
graphite	*R*_int_ = 0.022
Detector resolution: 10.5081 pixels mm^-1^	θ_max_ = 32.7°, θ_min_ = 4.8°
φ and ω scans	*h* = −10→12
Absorption correction: multi-scan (*CrysAlis RED*; Oxford Diffraction, 2007)	*k* = −12→14
*T*_min_ = 0.910, *T*_max_ = 0.972	*l* = −25→26
18429 measured reflections	

### Refinement

**Table:**

Refinement on *F*^2^	Primary atom site location: structure-invariant direct methods
Least-squares matrix: full	Secondary atom site location: difference Fourier map
*R*[*F*^2^ > 2σ(*F*^2^)] = 0.046	Hydrogen site location: inferred from neighbouring sites
*wR*(*F*^2^) = 0.126	H-atom parameters constrained
*S* = 1.03	*w* = 1/[σ^2^(*F*_o_^2^) + (0.0682*P*)^2^] where *P* = (*F*_o_^2^ + 2*F*_c_^2^)/3
9328 reflections	(Δ/σ)_max_ = 0.001
397 parameters	Δρ_max_ = 0.39 e Å^−^^3^
24 restraints	Δρ_min_ = −0.31 e Å^−^^3^

### Special details

**Table:**

Geometry. All e.s.d.'s (except the e.s.d. in the dihedral angle between two l.s. planes) are estimated using the full covariance matrix. The cell e.s.d.'s are taken into account individually in the estimation of e.s.d.'s in distances, angles and torsion angles; correlations between e.s.d.'s in cell parameters are only used when they are defined by crystal symmetry. An approximate (isotropic) treatment of cell e.s.d.'s is used for estimating e.s.d.'s involving l.s. planes.
Refinement. Refinement of *F*^2^ against ALL reflections. The weighted *R*-factor *wR* and goodness of fit *S* are based on *F*^2^, conventional *R*-factors *R* are based on *F*, with *F* set to zero for negative *F*^2^. The threshold expression of *F*^2^ > σ(*F*^2^) is used only for calculating *R*-factors(gt) *etc*. and is not relevant to the choice of reflections for refinement. *R*-factors based on *F*^2^ are statistically about twice as large as those based on *F*, and *R*- factors based on ALL data will be even larger.

### Fractional atomic coordinates and isotropic or equivalent isotropic displacement parameters (Å^2^)

**Table:**

	*x*	*y*	*z*	*U*_iso_*/*U*_eq_	Occ. (<1)
O1A	0.10752 (10)	0.23733 (10)	0.53343 (5)	0.02465 (19)	
O2A	0.12422 (13)	0.08237 (12)	0.64704 (6)	0.0414 (3)	
O3A	0.14903 (16)	−0.14166 (12)	0.61794 (7)	0.0549 (3)	
O4AA	0.4251 (3)	−0.2570 (3)	0.4160 (2)	0.0556 (8)	0.825 (10)
O5AA	0.4834 (4)	−0.0983 (5)	0.33859 (12)	0.0463 (8)	0.825 (10)
O4AB	0.4136 (17)	−0.2763 (15)	0.4447 (10)	0.0556 (8)	0.175 (10)
O5AB	0.4388 (16)	−0.1576 (18)	0.3475 (7)	0.0463 (8)	0.175 (10)
O6A	0.19712 (11)	0.28535 (11)	0.33215 (5)	0.0302 (2)	
O7A	0.24781 (13)	0.42205 (10)	0.44357 (5)	0.0365 (2)	
N1A	0.15738 (14)	−0.01478 (13)	0.60401 (7)	0.0341 (3)	
N2A	0.41627 (16)	−0.14450 (15)	0.39320 (9)	0.0470 (4)	
N3A	0.22611 (12)	0.30132 (12)	0.40164 (6)	0.0234 (2)	
C1A	0.18060 (13)	0.15168 (13)	0.50522 (6)	0.0197 (2)	
C2A	0.20815 (15)	0.02039 (13)	0.53354 (7)	0.0251 (3)	
C3A	0.27856 (16)	−0.07744 (14)	0.49621 (8)	0.0318 (3)	
H3AA	0.2900	−0.1645	0.5161	0.038*	
C4A	0.33233 (15)	−0.04861 (14)	0.42997 (8)	0.0301 (3)	
C5A	0.31226 (14)	0.07552 (14)	0.39838 (7)	0.0245 (3)	
H5AA	0.3472	0.0938	0.3522	0.029*	
C6A	0.24091 (14)	0.17030 (13)	0.43558 (6)	0.0193 (2)	
O1B	0.60822 (9)	0.83673 (9)	0.95805 (4)	0.01743 (16)	
O2B	0.65404 (10)	0.61445 (9)	0.85595 (5)	0.02139 (18)	
O3B	0.49768 (9)	0.66911 (8)	0.76153 (4)	0.01681 (16)	
N1B	0.14290 (11)	0.41809 (11)	0.66309 (5)	0.0195 (2)	
H1BD	0.1429	0.3307	0.6313	0.023*	
C1B	0.7672 (2)	0.65985 (16)	1.04798 (8)	0.0374 (3)	
H1BA	0.6537	0.6194	1.0317	0.056*	
H1BB	0.7985	0.6305	1.0966	0.056*	
H1BC	0.8215	0.6193	1.0099	0.056*	
C2B	0.81046 (16)	0.83003 (14)	1.05690 (7)	0.0266 (3)	
H2BA	0.9237	0.8703	1.0776	0.032*	
H2BB	0.7522	0.8696	1.0941	0.032*	
C3B	0.77559 (13)	0.88685 (13)	0.98354 (6)	0.0199 (2)	
H3BA	0.8118	0.9984	0.9919	0.024*	
H3BB	0.8303	0.8461	0.9450	0.024*	
C4B	0.55382 (13)	0.83591 (12)	0.88023 (6)	0.0144 (2)	
C5B	0.37558 (13)	0.80952 (12)	0.87150 (6)	0.0161 (2)	
C6B	0.29341 (14)	0.72390 (13)	0.91802 (6)	0.0210 (2)	
H6BA	0.3488	0.6901	0.9578	0.025*	
C7B	0.13033 (15)	0.68740 (15)	0.90661 (7)	0.0270 (3)	
H7BA	0.0751	0.6295	0.9389	0.032*	
C8B	0.04823 (15)	0.73485 (14)	0.84858 (7)	0.0273 (3)	
H8BA	−0.0630	0.7092	0.8408	0.033*	
C9B	0.12932 (14)	0.82027 (14)	0.80174 (7)	0.0252 (3)	
H9BA	0.0734	0.8531	0.7618	0.030*	
C10B	0.29213 (14)	0.85778 (13)	0.81321 (6)	0.0195 (2)	
H10A	0.3470	0.9167	0.7812	0.023*	
C11B	0.63706 (13)	0.98164 (12)	0.85584 (6)	0.0154 (2)	
C12B	0.60946 (14)	1.11664 (13)	0.88757 (6)	0.0211 (2)	
H12A	0.5343	1.1146	0.9202	0.025*	
C13B	0.69100 (16)	1.25303 (13)	0.87166 (7)	0.0256 (3)	
H13A	0.6711	1.3440	0.8932	0.031*	
C14B	0.80181 (16)	1.25757 (14)	0.82429 (7)	0.0272 (3)	
H14A	0.8573	1.3513	0.8133	0.033*	
C15B	0.83122 (15)	1.12444 (14)	0.79297 (7)	0.0239 (2)	
H15A	0.9075	1.1270	0.7609	0.029*	
C16B	0.74833 (13)	0.98724 (12)	0.80886 (6)	0.0178 (2)	
H16A	0.7683	0.8964	0.7872	0.021*	
C17B	0.57857 (12)	0.69440 (12)	0.83240 (6)	0.0154 (2)	
C18B	0.48344 (13)	0.52459 (12)	0.71359 (6)	0.0174 (2)	
H18A	0.5894	0.5082	0.7114	0.021*	
C19B	0.38094 (14)	0.39610 (12)	0.74338 (7)	0.0199 (2)	
H19A	0.3784	0.2990	0.7124	0.024*	
H19B	0.4277	0.3959	0.7962	0.024*	
C20B	0.21450 (14)	0.40916 (13)	0.74143 (6)	0.0208 (2)	
H20A	0.1506	0.3204	0.7583	0.025*	
H20B	0.2153	0.5006	0.7767	0.025*	
C21B	−0.02218 (15)	0.42444 (16)	0.65909 (8)	0.0303 (3)	
H21A	−0.0258	0.5157	0.6921	0.045*	
H21B	−0.0833	0.3353	0.6758	0.045*	
H21C	−0.0665	0.4264	0.6068	0.045*	
C22B	0.23950 (15)	0.54977 (13)	0.63493 (7)	0.0216 (2)	
H22A	0.2411	0.6449	0.6675	0.026*	
H22B	0.1915	0.5522	0.5827	0.026*	
C23B	0.40595 (14)	0.53774 (13)	0.63573 (6)	0.0208 (2)	
H23A	0.4686	0.6280	0.6195	0.025*	
H23B	0.4045	0.4482	0.5989	0.025*	

### Atomic displacement parameters (Å^2^)

**Table:**

	*U*^11^	*U*^22^	*U*^33^	*U*^12^	*U*^13^	*U*^23^
O1A	0.0261 (5)	0.0273 (5)	0.0207 (4)	0.0103 (4)	0.0027 (3)	0.0001 (3)
O2A	0.0526 (7)	0.0354 (6)	0.0290 (5)	−0.0030 (5)	0.0071 (5)	0.0090 (4)
O3A	0.0755 (9)	0.0329 (6)	0.0510 (7)	0.0041 (6)	−0.0062 (6)	0.0252 (5)
O4AA	0.0423 (8)	0.0202 (8)	0.102 (2)	0.0160 (7)	0.0016 (12)	−0.0015 (11)
O5AA	0.0380 (12)	0.0642 (18)	0.0363 (8)	0.0307 (12)	−0.0025 (7)	−0.0156 (9)
O4AB	0.0423 (8)	0.0202 (8)	0.102 (2)	0.0160 (7)	0.0016 (12)	−0.0015 (11)
O5AB	0.0380 (12)	0.0642 (18)	0.0363 (8)	0.0307 (12)	−0.0025 (7)	−0.0156 (9)
O6A	0.0272 (5)	0.0441 (6)	0.0212 (4)	0.0108 (4)	0.0046 (3)	0.0097 (4)
O7A	0.0551 (7)	0.0220 (5)	0.0306 (5)	0.0106 (4)	0.0009 (4)	0.0038 (4)
N1A	0.0361 (7)	0.0246 (6)	0.0310 (6)	−0.0054 (5)	−0.0104 (5)	0.0102 (5)
N2A	0.0293 (7)	0.0380 (8)	0.0613 (10)	0.0194 (6)	−0.0196 (6)	−0.0268 (7)
N3A	0.0211 (5)	0.0263 (5)	0.0227 (5)	0.0065 (4)	0.0027 (4)	0.0047 (4)
C1A	0.0157 (6)	0.0188 (5)	0.0193 (5)	0.0003 (4)	−0.0028 (4)	−0.0005 (4)
C2A	0.0237 (6)	0.0188 (6)	0.0262 (6)	−0.0017 (5)	−0.0060 (5)	0.0046 (5)
C3A	0.0273 (7)	0.0151 (6)	0.0443 (8)	0.0029 (5)	−0.0157 (6)	0.0011 (5)
C4A	0.0218 (7)	0.0225 (6)	0.0391 (7)	0.0102 (5)	−0.0103 (5)	−0.0119 (5)
C5A	0.0166 (6)	0.0296 (6)	0.0233 (6)	0.0075 (5)	−0.0028 (4)	−0.0060 (5)
C6A	0.0167 (6)	0.0183 (5)	0.0206 (5)	0.0051 (4)	−0.0018 (4)	−0.0003 (4)
O1B	0.0139 (4)	0.0227 (4)	0.0145 (4)	0.0038 (3)	0.0002 (3)	0.0038 (3)
O2B	0.0182 (4)	0.0180 (4)	0.0274 (4)	0.0073 (3)	−0.0006 (3)	0.0019 (3)
O3B	0.0179 (4)	0.0145 (4)	0.0164 (4)	0.0042 (3)	0.0014 (3)	−0.0011 (3)
N1B	0.0163 (5)	0.0198 (5)	0.0207 (5)	0.0043 (4)	0.0015 (4)	−0.0001 (4)
C1B	0.0476 (10)	0.0365 (8)	0.0302 (7)	0.0140 (7)	0.0002 (6)	0.0141 (6)
C2B	0.0267 (7)	0.0324 (7)	0.0197 (6)	0.0106 (5)	−0.0027 (5)	0.0024 (5)
C3B	0.0154 (6)	0.0198 (5)	0.0214 (5)	0.0028 (4)	−0.0035 (4)	0.0023 (4)
C4B	0.0133 (5)	0.0153 (5)	0.0140 (5)	0.0038 (4)	0.0010 (4)	0.0018 (4)
C5B	0.0141 (5)	0.0168 (5)	0.0159 (5)	0.0040 (4)	0.0022 (4)	−0.0019 (4)
C6B	0.0179 (6)	0.0241 (6)	0.0201 (5)	0.0043 (4)	0.0050 (4)	0.0020 (4)
C7B	0.0189 (6)	0.0306 (7)	0.0296 (6)	0.0020 (5)	0.0104 (5)	0.0018 (5)
C8B	0.0128 (6)	0.0315 (7)	0.0341 (7)	0.0058 (5)	0.0040 (5)	−0.0059 (5)
C9B	0.0180 (6)	0.0293 (6)	0.0271 (6)	0.0110 (5)	−0.0017 (4)	−0.0027 (5)
C10B	0.0170 (6)	0.0208 (5)	0.0206 (5)	0.0067 (4)	0.0025 (4)	0.0015 (4)
C11B	0.0133 (5)	0.0157 (5)	0.0155 (5)	0.0028 (4)	−0.0005 (4)	0.0022 (4)
C12B	0.0230 (6)	0.0185 (5)	0.0219 (6)	0.0068 (4)	0.0037 (4)	0.0012 (4)
C13B	0.0323 (7)	0.0151 (5)	0.0268 (6)	0.0055 (5)	0.0003 (5)	0.0006 (4)
C14B	0.0324 (7)	0.0186 (6)	0.0248 (6)	−0.0027 (5)	−0.0005 (5)	0.0069 (5)
C15B	0.0230 (6)	0.0254 (6)	0.0203 (6)	0.0000 (5)	0.0043 (4)	0.0051 (4)
C16B	0.0175 (6)	0.0170 (5)	0.0173 (5)	0.0029 (4)	0.0018 (4)	0.0017 (4)
C17B	0.0112 (5)	0.0142 (5)	0.0192 (5)	0.0008 (4)	0.0034 (4)	0.0018 (4)
C18B	0.0156 (5)	0.0144 (5)	0.0202 (5)	0.0036 (4)	0.0031 (4)	−0.0030 (4)
C19B	0.0185 (6)	0.0148 (5)	0.0239 (6)	0.0025 (4)	0.0002 (4)	0.0023 (4)
C20B	0.0184 (6)	0.0209 (6)	0.0208 (5)	0.0011 (4)	0.0035 (4)	0.0039 (4)
C21B	0.0181 (6)	0.0378 (7)	0.0335 (7)	0.0093 (5)	0.0012 (5)	0.0008 (6)
C22B	0.0249 (6)	0.0194 (5)	0.0194 (5)	0.0055 (5)	0.0010 (4)	0.0030 (4)
C23B	0.0228 (6)	0.0193 (5)	0.0175 (5)	0.0006 (4)	0.0062 (4)	−0.0002 (4)

### Geometric parameters (Å, °)

**Table:**

O1A—C1A	1.2477 (14)	C4B—C17B	1.5543 (14)
O2A—N1A	1.2302 (16)	C5B—C6B	1.3901 (16)
O3A—N1A	1.2241 (15)	C5B—C10B	1.3962 (15)
O4AA—N2A	1.190 (4)	C6B—C7B	1.3927 (17)
O5AA—N2A	1.289 (4)	C6B—H6BA	0.9500
O4AB—N2A	1.632 (16)	C7B—C8B	1.3825 (19)
O5AB—N2A	0.876 (12)	C7B—H7BA	0.9500
O6A—N3A	1.2295 (12)	C8B—C9B	1.3891 (19)
O7A—N3A	1.2235 (13)	C8B—H8BA	0.9500
N1A—C2A	1.4580 (18)	C9B—C10B	1.3900 (17)
N2A—C4A	1.4501 (18)	C9B—H9BA	0.9500
N3A—C6A	1.4601 (15)	C10B—H10A	0.9500
C1A—C2A	1.4461 (17)	C11B—C16B	1.3869 (16)
C1A—C6A	1.4481 (16)	C11B—C12B	1.4004 (15)
C2A—C3A	1.3817 (19)	C12B—C13B	1.3840 (17)
C3A—C4A	1.382 (2)	C12B—H12A	0.9500
C3A—H3AA	0.9500	C13B—C14B	1.3892 (19)
C4A—C5A	1.3921 (19)	C13B—H13A	0.9500
C5A—C6A	1.3665 (16)	C14B—C15B	1.3901 (18)
C5A—H5AA	0.9500	C14B—H14A	0.9500
O1B—C4B	1.4243 (12)	C15B—C16B	1.3939 (16)
O1B—C3B	1.4431 (14)	C15B—H15A	0.9500
O2B—C17B	1.2013 (13)	C16B—H16A	0.9500
O3B—C17B	1.3450 (12)	C18B—C23B	1.5180 (16)
O3B—C18B	1.4647 (12)	C18B—C19B	1.5217 (16)
N1B—C21B	1.4838 (16)	C18B—H18A	1.0000
N1B—C20B	1.4961 (14)	C19B—C20B	1.5183 (17)
N1B—C22B	1.5039 (15)	C19B—H19A	0.9900
N1B—H1BD	0.9300	C19B—H19B	0.9900
C1B—C2B	1.5126 (19)	C20B—H20A	0.9900
C1B—H1BA	0.9800	C20B—H20B	0.9900
C1B—H1BB	0.9800	C21B—H21A	0.9800
C1B—H1BC	0.9800	C21B—H21B	0.9800
C2B—C3B	1.5140 (16)	C21B—H21C	0.9800
C2B—H2BA	0.9900	C22B—C23B	1.5175 (17)
C2B—H2BB	0.9900	C22B—H22A	0.9900
C3B—H3BA	0.9900	C22B—H22B	0.9900
C3B—H3BB	0.9900	C23B—H23A	0.9900
C4B—C11B	1.5259 (15)	C23B—H23B	0.9900
C4B—C5B	1.5339 (15)		
			
O3A—N1A—O2A	122.35 (13)	C7B—C6B—H6BA	119.8
O3A—N1A—C2A	118.08 (13)	C8B—C7B—C6B	120.44 (12)
O2A—N1A—C2A	119.58 (11)	C8B—C7B—H7BA	119.8
O5AB—N2A—O4AA	104.2 (9)	C6B—C7B—H7BA	119.8
O5AB—N2A—O5AA	27.2 (10)	C7B—C8B—C9B	119.64 (11)
O4AA—N2A—O5AA	123.4 (2)	C7B—C8B—H8BA	120.2
O5AB—N2A—C4A	133.0 (8)	C9B—C8B—H8BA	120.2
O4AA—N2A—C4A	119.5 (3)	C8B—C9B—C10B	120.12 (11)
O5AA—N2A—C4A	117.02 (17)	C8B—C9B—H9BA	119.9
O5AB—N2A—O4AB	119.9 (9)	C10B—C9B—H9BA	119.9
O4AA—N2A—O4AB	15.8 (6)	C9B—C10B—C5B	120.47 (11)
O5AA—N2A—O4AB	138.1 (5)	C9B—C10B—H10A	119.8
C4A—N2A—O4AB	104.3 (6)	C5B—C10B—H10A	119.8
O7A—N3A—O6A	123.43 (10)	C16B—C11B—C12B	118.87 (10)
O7A—N3A—C6A	118.42 (10)	C16B—C11B—C4B	122.62 (9)
O6A—N3A—C6A	118.10 (10)	C12B—C11B—C4B	118.27 (10)
O1A—C1A—C2A	125.93 (11)	C13B—C12B—C11B	120.43 (11)
O1A—C1A—C6A	122.04 (10)	C13B—C12B—H12A	119.8
C2A—C1A—C6A	111.94 (10)	C11B—C12B—H12A	119.8
C3A—C2A—C1A	123.20 (12)	C12B—C13B—C14B	120.32 (11)
C3A—C2A—N1A	117.22 (11)	C12B—C13B—H13A	119.8
C1A—C2A—N1A	119.56 (12)	C14B—C13B—H13A	119.8
C2A—C3A—C4A	120.02 (12)	C13B—C14B—C15B	119.78 (11)
C2A—C3A—H3AA	120.0	C13B—C14B—H14A	120.1
C4A—C3A—H3AA	120.0	C15B—C14B—H14A	120.1
C3A—C4A—C5A	121.07 (12)	C14B—C15B—C16B	119.72 (11)
C3A—C4A—N2A	120.50 (13)	C14B—C15B—H15A	120.1
C5A—C4A—N2A	118.39 (14)	C16B—C15B—H15A	120.1
C6A—C5A—C4A	118.15 (12)	C11B—C16B—C15B	120.87 (10)
C6A—C5A—H5AA	120.9	C11B—C16B—H16A	119.6
C4A—C5A—H5AA	120.9	C15B—C16B—H16A	119.6
C5A—C6A—C1A	125.55 (11)	O2B—C17B—O3B	124.27 (10)
C5A—C6A—N3A	116.38 (11)	O2B—C17B—C4B	125.00 (10)
C1A—C6A—N3A	118.07 (10)	O3B—C17B—C4B	110.66 (8)
C4B—O1B—C3B	116.70 (8)	O3B—C18B—C23B	105.04 (8)
C17B—O3B—C18B	117.48 (8)	O3B—C18B—C19B	110.30 (9)
C21B—N1B—C20B	111.66 (9)	C23B—C18B—C19B	110.25 (9)
C21B—N1B—C22B	111.21 (9)	O3B—C18B—H18A	110.4
C20B—N1B—C22B	110.85 (9)	C23B—C18B—H18A	110.4
C21B—N1B—H1BD	107.6	C19B—C18B—H18A	110.4
C20B—N1B—H1BD	107.6	C20B—C19B—C18B	112.27 (9)
C22B—N1B—H1BD	107.6	C20B—C19B—H19A	109.1
C2B—C1B—H1BA	109.5	C18B—C19B—H19A	109.1
C2B—C1B—H1BB	109.5	C20B—C19B—H19B	109.1
H1BA—C1B—H1BB	109.5	C18B—C19B—H19B	109.1
C2B—C1B—H1BC	109.5	H19A—C19B—H19B	107.9
H1BA—C1B—H1BC	109.5	N1B—C20B—C19B	110.68 (9)
H1BB—C1B—H1BC	109.5	N1B—C20B—H20A	109.5
C1B—C2B—C3B	113.52 (10)	C19B—C20B—H20A	109.5
C1B—C2B—H2BA	108.9	N1B—C20B—H20B	109.5
C3B—C2B—H2BA	108.9	C19B—C20B—H20B	109.5
C1B—C2B—H2BB	108.9	H20A—C20B—H20B	108.1
C3B—C2B—H2BB	108.9	N1B—C21B—H21A	109.5
H2BA—C2B—H2BB	107.7	N1B—C21B—H21B	109.5
O1B—C3B—C2B	107.54 (10)	H21A—C21B—H21B	109.5
O1B—C3B—H3BA	110.2	N1B—C21B—H21C	109.5
C2B—C3B—H3BA	110.2	H21A—C21B—H21C	109.5
O1B—C3B—H3BB	110.2	H21B—C21B—H21C	109.5
C2B—C3B—H3BB	110.2	N1B—C22B—C23B	110.62 (9)
H3BA—C3B—H3BB	108.5	N1B—C22B—H22A	109.5
O1B—C4B—C11B	110.90 (8)	C23B—C22B—H22A	109.5
O1B—C4B—C5B	106.28 (8)	N1B—C22B—H22B	109.5
C11B—C4B—C5B	113.50 (8)	C23B—C22B—H22B	109.5
O1B—C4B—C17B	108.42 (8)	H22A—C22B—H22B	108.1
C11B—C4B—C17B	112.01 (9)	C22B—C23B—C18B	112.20 (9)
C5B—C4B—C17B	105.37 (8)	C22B—C23B—H23A	109.2
C6B—C5B—C10B	119.00 (10)	C18B—C23B—H23A	109.2
C6B—C5B—C4B	119.43 (10)	C22B—C23B—H23B	109.2
C10B—C5B—C4B	121.29 (10)	C18B—C23B—H23B	109.2
C5B—C6B—C7B	120.33 (11)	H23A—C23B—H23B	107.9
C5B—C6B—H6BA	119.8		
			
O1A—C1A—C2A—C3A	174.36 (11)	C10B—C5B—C6B—C7B	−0.18 (16)
C6A—C1A—C2A—C3A	−2.28 (16)	C4B—C5B—C6B—C7B	−174.30 (10)
O1A—C1A—C2A—N1A	−4.32 (18)	C5B—C6B—C7B—C8B	0.50 (18)
C6A—C1A—C2A—N1A	179.04 (10)	C6B—C7B—C8B—C9B	−0.40 (18)
O3A—N1A—C2A—C3A	−15.05 (17)	C7B—C8B—C9B—C10B	−0.01 (18)
O2A—N1A—C2A—C3A	165.15 (12)	C8B—C9B—C10B—C5B	0.32 (17)
O3A—N1A—C2A—C1A	163.71 (12)	C6B—C5B—C10B—C9B	−0.22 (16)
O2A—N1A—C2A—C1A	−16.09 (17)	C4B—C5B—C10B—C9B	173.79 (10)
C1A—C2A—C3A—C4A	2.68 (19)	O1B—C4B—C11B—C16B	107.93 (11)
N1A—C2A—C3A—C4A	−178.61 (11)	C5B—C4B—C11B—C16B	−132.52 (10)
C2A—C3A—C4A—C5A	−2.15 (19)	C17B—C4B—C11B—C16B	−13.34 (13)
C2A—C3A—C4A—N2A	175.61 (11)	O1B—C4B—C11B—C12B	−66.41 (12)
O5AB—N2A—C4A—C3A	163.1 (14)	C5B—C4B—C11B—C12B	53.14 (12)
O4AA—N2A—C4A—C3A	7.5 (2)	C17B—C4B—C11B—C12B	172.32 (9)
O5AA—N2A—C4A—C3A	−169.72 (17)	C16B—C11B—C12B—C13B	0.58 (16)
O4AB—N2A—C4A—C3A	3.0 (6)	C4B—C11B—C12B—C13B	175.13 (10)
O5AB—N2A—C4A—C5A	−19.1 (15)	C11B—C12B—C13B—C14B	−0.33 (18)
O4AA—N2A—C4A—C5A	−174.66 (18)	C12B—C13B—C14B—C15B	−0.23 (18)
O5AA—N2A—C4A—C5A	8.1 (2)	C13B—C14B—C15B—C16B	0.52 (18)
O4AB—N2A—C4A—C5A	−179.2 (5)	C12B—C11B—C16B—C15B	−0.28 (16)
C3A—C4A—C5A—C6A	1.44 (18)	C4B—C11B—C16B—C15B	−174.59 (10)
N2A—C4A—C5A—C6A	−176.37 (11)	C14B—C15B—C16B—C11B	−0.26 (17)
C4A—C5A—C6A—C1A	−1.26 (18)	C18B—O3B—C17B—O2B	8.72 (16)
C4A—C5A—C6A—N3A	178.35 (11)	C18B—O3B—C17B—C4B	−168.41 (9)
O1A—C1A—C6A—C5A	−175.18 (11)	O1B—C4B—C17B—O2B	−11.26 (15)
C2A—C1A—C6A—C5A	1.61 (16)	C11B—C4B—C17B—O2B	111.44 (12)
O1A—C1A—C6A—N3A	5.21 (16)	C5B—C4B—C17B—O2B	−124.70 (11)
C2A—C1A—C6A—N3A	−178.00 (10)	O1B—C4B—C17B—O3B	165.86 (8)
O7A—N3A—C6A—C5A	−140.18 (12)	C11B—C4B—C17B—O3B	−71.45 (11)
O6A—N3A—C6A—C5A	37.65 (15)	C5B—C4B—C17B—O3B	52.41 (11)
O7A—N3A—C6A—C1A	39.47 (16)	C17B—O3B—C18B—C23B	−172.99 (9)
O6A—N3A—C6A—C1A	−142.70 (11)	C17B—O3B—C18B—C19B	68.22 (12)
C4B—O1B—C3B—C2B	−161.04 (9)	O3B—C18B—C19B—C20B	62.58 (12)
C1B—C2B—C3B—O1B	63.23 (14)	C23B—C18B—C19B—C20B	−52.98 (12)
C3B—O1B—C4B—C11B	−45.26 (11)	C21B—N1B—C20B—C19B	177.60 (9)
C3B—O1B—C4B—C5B	−169.05 (8)	C22B—N1B—C20B—C19B	−57.80 (12)
C3B—O1B—C4B—C17B	78.11 (11)	C18B—C19B—C20B—N1B	55.83 (12)
O1B—C4B—C5B—C6B	−30.91 (13)	C21B—N1B—C22B—C23B	−177.23 (9)
C11B—C4B—C5B—C6B	−153.07 (10)	C20B—N1B—C22B—C23B	57.92 (12)
C17B—C4B—C5B—C6B	84.02 (11)	N1B—C22B—C23B—C18B	−55.98 (12)
O1B—C4B—C5B—C10B	155.10 (9)	O3B—C18B—C23B—C22B	−65.73 (11)
C11B—C4B—C5B—C10B	32.95 (13)	C19B—C18B—C23B—C22B	53.09 (12)
C17B—C4B—C5B—C10B	−89.96 (11)		

### Hydrogen-bond geometry (Å, °)

**Table:**

*D*—H···*A*	*D*—H	H···*A*	*D*···*A*	*D*—H···*A*
N1B—H1BD···O1A	0.93	1.81	2.6276 (12)	145
N1B—H1BD···O2A	0.93	2.33	3.0537 (15)	135
C20B—H20B···Cg2	0.99	2.77	3.7553 (13)	173
